# Glycogen synthase kinase-3β inactivation inhibits tumor necrosis factor-α production in microglia by modulating nuclear factor κB and MLK3/JNK signaling cascades

**DOI:** 10.1186/1742-2094-7-99

**Published:** 2010-12-31

**Authors:** Mei-Jen Wang, Hsin-Yi Huang, Wu-Fu Chen, Hui-Fen Chang, Jon-Son Kuo

**Affiliations:** 1 Department of Medical Research, Neuro-Medical Scientific Center, Buddhist Tzu Chi General Hospital, Hualien 970, Taiwan; 2 Institute of Medical Sciences, Buddhist Tzu Chi University, Hualien 970, Taiwan; 3 Department of Neurosurgery, Chang Gung Memorial Hospital- Kaohsiung Medical Center, Chang Gung University College of Medicine, Kaohsiung, Taiwan; 4 Department of Marine Biotechnology and Resources, National Sun Yat-Sen University, Kaohsiung, Taiwan

## Abstract

**Background:**

Deciphering the mechanisms that modulate the inflammatory response induced by microglial activation not only improves our insight into neuroinflammation but also provides avenues for designing novel therapies that could halt inflammation-induced neuronal degeneration. Decreasing glycogen synthase kinase-3β (GSK-3β) activity has therapeutic benefits in inflammatory diseases. However, the exact molecular mechanisms underlying GSK-3β inactivation-mediated suppression of the inflammatory response induced by microglial activation have not been completely clarified. Tumor necrosis factor-α (TNF-α) plays a central role in injury caused by neuroinflammation. We investigated the regulatory effect of GSK-3β on TNF-α production by microglia to discern the molecular mechanisms of this modulation.

**Methods:**

Lipopolysaccharide (LPS) was used to induce an inflammatory response in cultured primary microglia or murine BV-2 microglial cells. Release of TNF-α was measured by ELISA. Signaling molecules were analyzed by western blotting, and activation of NF-κB and AP-1 was measured by ELISA-based DNA binding analysis and luciferase reporter assay. Protein interaction was examined by coimmunoprecipitation.

**Results:**

Inhibition of GSK-3β by selective GSK-3β inhibitors or by RNA interference attenuated LPS-induced TNF-α production in cultured microglia. Exploration of the mechanisms by which GSK-3β positively regulates inflammatory response showed that LPS-induced IκB-α degradation, NF-κBp65 nuclear translocation, and p65 DNA binding activity were not affected by inhibition of GSK-3β activity. However, GSK-3β inactivation inhibited transactivation activity of p65 by deacetylating p65 at lysine 310. Furthermore, we also demonstrated a functional interaction between mixed lineage kinase 3 (MLK3) and GSK-3β during LPS-induced TNF-α production in microglia. The phosphorylated levels of MLK3, MKK4, and JNK were increased upon LPS treatment. Decreasing GSK-3β activity blocked MLK3 signaling cascades through disruption of MLK3 dimerization-induced autophosphorylation, ultimately leading to a decrease in TNF-α secretion.

**Conclusion:**

These results suggest that inactivation of GSK-3β might represent a potential strategy to downregulate microglia-mediated inflammatory processes.

## Background

Inflammatory processes, involving a host of cytokines, have been shown to be associated with ongoing neuronal degeneration in several neurodegenerative diseases. Activation of glial cells such as microglia and astrocytes is a characteristic finding in brain inflammation. Microglia, as the immunocompetent resident cells of the brain, possess properties particularly suitable for mediating cellular inflammatory responses. The secretion of pro-inflammatory and neurotoxic mediators from activated microglia is believed to contribute to progressive damage in neurodegenerative disorders [1-3]. Therefore, deciphering the mechanisms that govern inflammation caused by microglial activation and its effects on brain are vital for understanding the pathogenesis of these diseases.

Glycogen synthase kinase 3 (GSK-3) is a multifunctional serine/threonine kinase found in all eukaryotes. There are two highly homologous mammalian isoforms of GSK-3, GSK-3α and GSK-3β. GSK-3β is a key regulator of numerous signaling pathways, and is involved in a wide range of cellular processes ranging from glycogen metabolism to the regulation of cell survival and neuronal polarity [4,5]. Furthermore, the function of GSK-3β in signaling mechanisms that activate nuclear factor κB (NF-κB), as well as the resulting effects on NF-κB-mediated gene expression, indicate that GSK-3β acts as a regulator of inflammation [6-8]. Although an effect of GSK-3β in modulation of inflammation has been identified, the potential role and mechanism for this effect are still controversial. Inhibition of GSK-3β by pharmacological inhibitors or by overexpression of a dominant negative mutant of GSK-3β enhances tumor necrosis factor-α (TNF-α) expression in lipopolysaccharide- (LPS-)stimulated cardiomyocytes [9]. Another report has implicated GSK-3β in inhibition of TNF-α- and interleukin- (IL-)1β-induced inflammatory gene expression [10]. Conversely, the identification of GSK-3β as a major regulator of peripheral inflammatory responses has shown that GSK-3β promotes the stimulus-induced production of several cytokines and the subsequent development of disease symptoms in animal models of inflammatory conditions [11]. Recently, GSK-3β inactivation has been shown to downregulate the inflammatory response induced by microglial activation [12,13]. However, the molecular mechanisms of downstream signal transduction leading to this anti-inflammatory effect of GSK-3β inhibition in microglia are not yet clearly understood.

TNF-α is a pro-inflammatory cytokine that is upregulated in the brain in response to various insults or injury. Activated microglia around an injured area have been shown to be the major source of this cytokine. Within the brain, inflammatory processes might be modulated by TNF-α through further activation of microglia and astrocytes [14,15]. TNF-α is known to induce generation of reactive oxygen intermediates associated with necrotic cell death, and it also induces changes in mitochondrial ultrastructure and function [16,17]. In addition, TNF-α also directly induces neuronal death by binding to TNF receptor 1 to trigger intracellular death-related signaling pathways [18]. Increased TNF-α production is seen in several neurodegenerative diseases and may contribute to secondary damage that further worsens a disease state [19-22]. For example, in Parkinson's disease (PD), significant increases in the expression of TNF-α and its receptors have been reported in the caudate and putamen of postmortem brain samples from patients with PD [20]. Several studies have demonstrated that blocking soluble TNF signaling attenuates loss of dopaminergic neurons in cellular and animal models of PD [23,24]. Moreover, increasing basic science, genetic, and clinical evidence now supports the concept that excess TNF-α plays a central role in Alzheimer's disease (AD) [19,25-27]. Administration of a TNF-α antagonist has been shown to improve cognition in AD patients [28], and to yield a mild increase in survival in a mouse model of amyotrophic lateral sclerosis [29]. Therefore, TNF-α can be considered to be a central mechanism of injury caused by neuroinflammation. The present study was initiated to evaluate the role of GSK-3β in the regulation of TNF-α production by microglia to discern the molecular mechanisms of this modulation.

## Methods

### Materials

LPS from *Escherichia coli *serotype O111:B4, AR-A014418, TWS119, TDZD, L803-mts, SP600125, K252a, BAY 11-7082 and pyrrolidinedithiocarbamate (PDTC) were obtained from Calbiochem (San Diego, CA). Cell culture ingredients were purchased from Invitrogen (Carlsbad, CA). Monoclonal mouse anti-transcription factor IIB (TFIIB) antibody was purchased from BD Biosciences (San Diego, CA). Polyclonal rabbit anti-phospho-Mixed lineage kinase 3 (MLK3) was from Upstate Biotechnology (Lake Placid, NY). Polyclonal rabbit anti-acetyl p65 and polyclonal rabbit anti-phospho-p65 (Ser276) were obtained from Abcam (Cambridge, UK). Antibodies against ERKs and phospho-ERKs were from Promega (Madison, WI). All other antibodies were from Cell Signaling Technology (Beverly, MA). All other reagents were from Sigma-Aldrich (St. Louis, MO).

### Microglial cultures

Primary microglia were prepared from ventral mesencephalon of 1-day-old Sprague-Dawley (SD) rats as previously described [30]. Briefly, ventral mesencephalic tissues, devoid of meninges and blood vessels, were dissociated by a mild mechanical trituration. The isolated cells (5 × 10^7^) were seeded in 150-cm^2 ^culture flasks in Dulbecco's modified Eagle's medium (DMEM) containing 10% fetal bovine serum (FBS), 100 U/ml penicillin, and 100 μg/ml streptomycin. The cells were maintained at 37°C in a humidified atmosphere of 5% CO_2 _and 95% air. The medium were changed 4 days later. Upon reaching confluence (12-14 days), microglia were separated from astrocytes by shaking the flasks for 2 hr at 180 rpm. Detached cells were plated into 24-wells plate in DMEM supplemented with 10% FBS, 100 U/ml penicillin, and 100 μg/ml streptomycin at a density of 2.5 × 10^5 ^cells per well. After 2 h of incubation at 37°C, nonadherent cells were removed. The purity of microglia cultures was assessed by using OX-42 antibody and more than 95% of cells were stained positively. Cells were cultured for 2 days before treatment.

Murine BV-2 microglial cells were maintained in DMEM supplemented with 10% FBS, 100 U/ml penicillin and 100 μg/ml streptomycin at 37°C in a humidified incubator under 5% CO_2_. Confluent cultures were trypsanized. Cells were plated into 24-wells plate at a density of 2 × 10^5 ^cells per well and then incubated for 24 h before treatment.

### Real-time RT-PCR analysis

The level of TNF-α gene expression was quantified using real-time RT-PCR analysis. Briefly, total RNA was extracted from microglia cultures with a cold RNA extraction solution (Ultraspec RNA; Biotecx Lab. Inc., Houston, TX). Total RNA was reverse transcribed with M-MLV reverse transcriptase and oligo-dT primers (SuperScript™First-Strand Synthesis System, Invitrogen). The primer sequences are as follows: for mouse TNF-α, 5'-TTC TGT CTA CTG AAC TTC GGG GTG ATC GGT CC-3' and 5'-GTA TGA GAT AGC AAA TCG GCT GAC GGT GTG GG-3'; for mouse β-actin, 5'-GTG GGC CGC TCT AGG CAC CAA-3' and 5'-CTC TTT GAT GTC ACG CAC GAT TTC-3'; for rat TNF-α, 5'-CAG GGC AAT GAT CCC AAA GTA-3' and 5'-GCA GTC AGA TCA TCT TCT CGA-3'; for rat β-actin, 5'-TTG TAA CCA ACT GGG ACG ATA TGG-3' and 5'-GAT CTT GAT CTT CAT GGT GCT AGG-3'. The SYBR green DNA PCR kit (Applied Biosystems, Foster City, CA) was used for real-time PCR analysis. The relative differences in expression between groups were analyzed on the basis of cycle time (Ct) values normalized with β-actin.

### Preparation of whole cell and nuclear extracts

Cells cultured in 10-cm Petri-dishes were washed twice with ice-cold PBS and lysed in M-PER^® ^mammalian protein extraction reagent (Pierce, Rockford, IL) containing 5 mM sodium orthovanadate, 1 mM PMSF, 10 μg/ml aprotinin, 10 μg/ml leupeptin, and 5 μg/ml pepstain A. After incubation for 5 min, cell lysates were centrifuged and the supernatants were collected. Nuclear extracts were prepared by using the NE-PER^® ^nuclear and cytoplasmic extraction reagents (Pierce) as per the manufacturer's instructions. Protein concentration of samples was determined by Bradford assay (Bio-Rad, Hemel, Hempstead, UK), and aliquots were stored at -80°C.

### Immunoprecipitation

The cytosolic lysates (300 μg of protein) prepared in immunoprecipitation buffer (20 mM Tris, pH 7.5, 150 mM NaCl, 1 mM EDTA, 1 mM EGTA, 1% Triton X-100, 2.5 mM sodium pyrophosphate, 1 mM β-glycerolphosphate, 1 mM Na_3_VO_4_, 1 μg/ml leupeptin, 1 mM PMSF) were incubated with a goat anti-MLK3 antibody (Santa Cruz Biotechnology, Santa Cruz, CA) with gentle rocking overnight at 4°C. PureProteome™protein A magnetic beads (Millipore, Billerica, MA) were added (10 μl of suspension) and rotated for 3 h at 4°C. The beads were then washed five times with ice-cold immunoprecipitation buffer. The pellet was resuspended with 20 μl 3X SDS sample buffer (187.5 mM Tris, pH 6.8, 6% SDS, 30% glycerol, and 0.3% bromophenol blue) either with or without the reducing agent DTT (final concentration, 150 mM), and boiled for 5 min. The samples were resolved by sodium dodecyl sulphate-polyacrylamide (SDS-PAGE) gel electrophoresis and MLK3 or GSK-3β was detected with antibodies.

### Western blotting

Western blot analysis was carried out using antibodies against phosphorylated members of the MAP kinase family; the specific phosphorylated sites were: IκB-α (Ser32), NF-κB p65 (Ser276, Ser468 and Ser536), c-Jun (Ser63), MKK4 (Ser257/Thr261), MKK7 (Ser271/Thr275), and MLK3 (Thr277/Ser281); and an acetylated site of NF-κB p65 (Lys310). Antibodies active against all forms of each mentioned protein, TFIIB or β-actin were used as internal controls to determine loading efficiency. Protein samples containing 50 μg of protein were separated on 10% SDS-PAGE gels and transferred to immobilon polyvinylidene difluoride membranes (Millipore). The membranes were incubated in Tris-buffered saline/Tween (TBST) buffer (0.1 M Tris/HCl, pH 7.4, 0.9% NaCl, 0.1% Tween 20) supplemented with 5% dry skim milk for 1 h to block nonspecific binding. After rinsing with TBST buffer, they were incubated with primary antibodies. The membranes were washed twice with TBST buffer followed by incubation with appropriate streptavidin-horseradish peroxidase-conjugated secondary antibodies. The antigen-antibody complexes were detected by using a chemiluminescence detection system (ECL, Amersham, Berkshire, England). The intensity of the band was quantified with a densitometric analysis (GS-800 Calibrated Densitometer, Bio-Rad), and calculated as the optical density × area of band.

### ELISA-based DNA binding analysis

The DNA-binding activity of NF-κB and activated protein-1 (AP-1) was quantified by ELISA using EZ-Detect™NF-κB p65 and c-jun Transcription Factor Kits (Pierce), respectively, according to the manufacturer's instructions. Briefly, 5 μg of nuclear extract was incubated in 96-well plates coated with immobilized oligonucleotides containing a consensus binding site for NF-κB (5'-GGGACTTTCC-3') or AP-1 (5'-TGAGTCA-3') for 1 h at room temperature. Following three washes, primary antibodies specific to p65 (for detection of NF-κB) or c-Jun (for detection of AP-1 complexes) was added and incubated again at room temperature for 1 hour. Addition of secondary antibodies conjugated to horseradish peroxidase was performed prior to the quantification of NF-κB or AP-1 DNA-binding activity by measuring luminescence (Fluoroskan Ascent FL Luminometer, ThermoLabsystems, Fremont, CA). The specificity of DNA-binding activity was verified by performing competition assays, in which an excess amount of soluble oligonucleotides (40 pmoles), that contained either intact or mutant consensus binding sequences, was coincubated in the above described assays.

### Transient transfection and luciferase assay

Luciferase reporter plasmids (NF-κB-Luc and AP-1-Luc, Clontech Laboratories, Palo Alto, CA) were transfected into BV-2 cells using Lipofectamine 2000 (Invitrogen) according to the protocol of the manufacturer. At 24 h after transfection, cells were treated with LPS in the absence or presence of TWS119 or SP600125 for another 6 h. Luciferase activity of cell lysates was determined luminometrically by the dual-luciferase assay system (Promega) as specified by the manufacturer. Each transfection was performed in duplicate, and all experiments were repeated at least three times. Luciferase activity was normalized to the protein content of the extracts. Relative luciferase activity was determined to reflect transcriptional activity of NF-κB and AP-1, expressed as the fold increase relative to the activity of untreated controls.

### RNA interference

Murine GSK-3β was targeted with a small interfering RNA (siRNA) duplex supplied by SignalSilence^® ^GSK-3β siRNA kit (Cell Signaling), according to the protocol of the manufacturer. SignalSilence^® ^Control siRNA, a non-targeted negative control duplex, was used as a control. siRNA duplexes were transfected into BV-2 cells for 48 h followed by treatment with 100 ng/ml LPS for 6 h. Released TNF-α was measured by ELISA.

### TNF-α assay

Primary microglia and BV-2 microglia were stimulated with LPS in the absence or presence of GSK-3β inhibitors, and supernatants were collected and kept frozen in aliquots at -80°C until use. Release of TNF-α was measured with a commercial enzyme-linked immunosorbent assay (ELISA) kit from R&D Systems (Minneapolis, MN) according to the manufacturer's instructions.

### Statistical analysis

All data are expressed as the mean ± SEM. Data were analyzed by one-way analysis of variance (ANOVA) followed by Scheffe's test. A *p *value less than 0.05 was considered statistically significant.

## Results

### GSK-3β inhibition decreases TNF-α production in LPS-stimulated microglia

**Figure 1:**
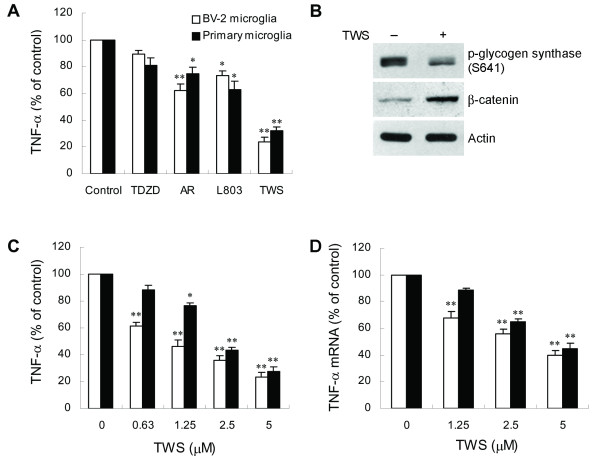
**GSK-3β inhibitors inhibit LPS-induced TNF-α production in microglia**. (A) Cells were preincubated with vehicle or GSK-3β inhibitors 10 μM TDZD-8 (TDZD), 10 μM AR-A014418 (AR), 10 μM L803-mts (L803), or 5 μM TWS119 (TWS) for 30 min, and subsequently treated with 100 ng/ml LPS for 6 h. Released TNF-α was measured by ELISA. Data are presented as mean ± SEM for three independent experiments. The TNF-α content in untreated cultures or cultures treated with inhibitor alone was not detectable. The levels of TNF-α in LPS-treated alone BV-2 cells and primary microglia were 3.78 ± 0.27 and 3.49 ± 0.22 ng/ml, respectively. (B) TWS119 inhibits activation of GSK-3β. BV-2 cells were exposed to DMSO or TWS119 (2.5 μM) for 60 min and analyzed by western blot, using the indicated specific antibodies. (C and D) Cells were pretreated with vehicle or various concentrations of TWS119 for 30 min followed by treatment with 100 ng/ml LPS for 6 h (C) or 2 h (D). Released TNF-α (C) is expressed as mean ± SEM for three independent experiments. The levels of TNF-α in LPS-treated alone BV-2 cells and primary microglia were 3.95 ± 0.32 and 3.62 ± 0.3 ng/ml, respectively. Expression of TNF-α mRNA (D) was quantified by real-time RT-PCR as described in *Methods*. The relative differences in expression between groups were analyzed on the basis of cycle time values normalized with β-actin. The relative differences between control and TWS119 pretreated groups were calculated and expressed as % of control. Data are presented as mean ± SEM for three independent experiments. *p < 0.05; **p < 0.01 compared with control.

Because TNF-α has been demonstrated to act as a central inflammatory mediator, we tested whether GSK-3β might modulate microglial activation by examining the effect of GSK-3β inhibitors on LPS-induced TNF-α release in microglia. Microglia were pretreated with four structurally distinct selective GSK-3β inhibitors, TDZD-8, AR-A014418, L803-mts or TWS119, for 30 min prior to stimulation with 100 ng/ml LPS. TDZD-8, AR-A014418 and L803-mts are specific GSK-3β inhibitors that selectively inhibit GSK-3β activity but do not significantly affect the activities of other protein kinases determined by in vitro kinase assays. GSK-3β has been shown to be the specific cellular target of TWS119, identified by affinity chromatography and by LC/MS, and is potently inhibited by this inhibitor [31]. As shown in Figure 1A, all GSK-3β inhibitors, except TDZD-8, significantly attenuated TNF-α production in response to LPS treatment. The GSK-3β inhibitors at the concentrations used in this study did not show cytotoxic effects. Among these inhibitors, TWS119 was shown to be the most potent inhibitor for reduction of TNF-α. We further confirmed the intracellular activity of TWS119 in inhibiting GSK-3β by examining phosphorylation of glycogen synthase and accumulation of β-catenin in TWS119-treated BV-2 cells. TWS119 effectively decreased the phosphorylation of glycogen synthase, and increased β-catenin levels (Figure 1B). Therefore, we used TWS119 to further determine the mechanism by which inactivation of GSK-3β downregulates LPS-induced TNF-α expression. Figure 1C shows that LPS-induced TNF-α release was robustly suppressed by TWS119 in a concentration-dependent manner. To investigate whether the reduction in TNF-α protein in microglia following a decrease in GSK-3β activity is due to suppression of TNF-α mRNA expression, real-time RT-PCR analysis was performed to assess the TNF-α mRNA levels. The results show that TWS119 treatment significantly reduced LPS-induced TNF-α mRNA expression in a dose-dependent manner (Figure 1D).

### Loss of endogenous GSK-3β decreases TNF-α production

**Figure 2:**
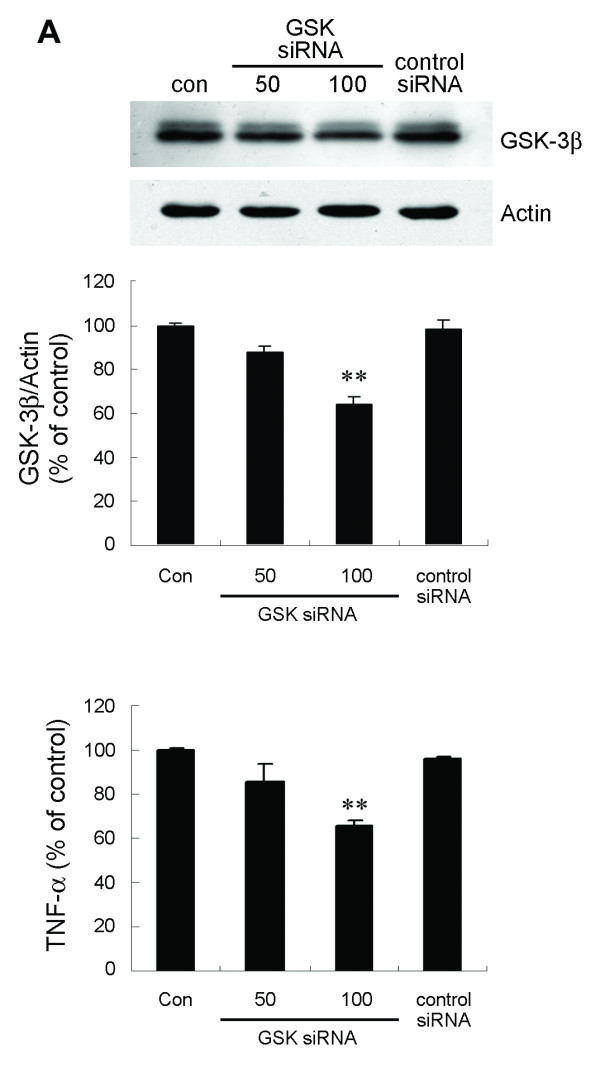
**siRNA targeting of GSK-3β inhibits TNF-α production by LPS-stimulated BV-2 cells**. (A) Cells were transfected with GSK-3β-specific (50 and 100 nM) or control siRNA (100 nM) duplexes for 48 h. Endogenous GSK-3β protein was analyzed by western blotting. (B) Parallel cultures were exposed to 100 ng/ml LPS for 6 h. Released TNF-α was measured by ELISA. Data are presented as mean ± SEM for three independent experiments. *p < 0.05; **p < 0.01 compared with control siRNA-transfected cultures. The level of TNF-α in control cultures was 6.08 ± 0.41 ng/ml.

To further assess the role of endogenous GSK-3β in LPS-induced TNF-α production, we downregulated GSK-3β expression using an RNA interference approach. BV-2 cells were transfected with GSK-3β-specific siRNA duplexes and, 48 h later, were exposed to LPS. After an additional 6 h, TNF-α content in the supernatant was determined. There was no effect on viability of the cultures following incubation with siRNA for 48 h. Downregulation of GSK-3β (Figure 2A) significantly reduced TNF-α release compared with control siRNA-treated cells (Figure 2B).

### IκB-α degradation is not regulated by GSK-3β

**Figure 3:**
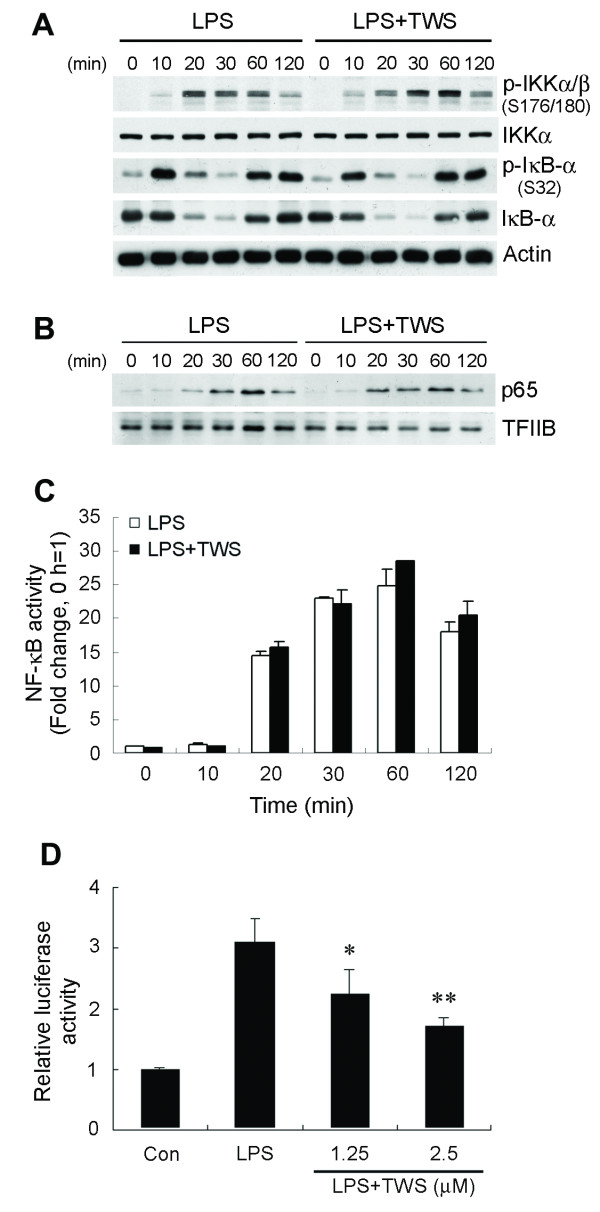
**Blockade of GSK-3β decreases NF-κB transcriptional activity**. BV-2 cells were preincubated with vehicle or TWS119 (2.5 μM) for 30 min before stimulation with 100 ng/ml LPS for the various times indicated. Whole cell lysates and nuclear extracts were prepared. (A) IκB-α degradation is not regulated by GSK-3β. Western analysis was used to determine total and phosphorylated IKKα/β and IκB-α proteins in whole cell extracts. (B) Localization of p65 to the nucleus was determined by immunoblotting. TFIIB immunoblotting was performed to monitor loading. (C) ELISA-based measurement of p65 DNA binding was analyzed as described in *Methods*. (D) Cells were transfected with a 3XNF-κB-luciferase reporter construct. 24 h post-transfection, cells were preincubated with vehicle or TWS119 (2.5 μM) for 30 min before stimulation with 100 ng/ml LPS for 6 h. Luciferase activity is presented as a fold of control. Data are presented as mean ± SEM for three independent experiments. *p < 0.05; **p < 0.01 compared with LPS alone.

We next investigated the molecular mechanisms involved in the GSK-3β inhibition-mediated reduction of TNF-α secretion seen in LPS-activated microglia. Clearly, NF-κB appears to be essential for maximal cytokine transcription after LPS stimulation. To determine whether GSK-3β inhibition influences LPS-induced NF-κB activation, we assessed the effect of TWS119 at different levels of the NF-κB signaling cascade after LPS stimulation. Activation of the IκB kinase (IKK) complex depends on phosphorylation, and has been demonstrated to be critical for NF-κB activation. After phosphorylation of IκB-α by the IKK complex, IκB-α is degraded and releases NF-κB, which translocates to the nucleus. Therefore, we first determined the effect of TWS119 on IκB-α phosphorylation and degradation. BV-2 cells were treated with TWS119 for 30 min prior to addition of LPS. As shown in Figure 3A, LPS- elicited IKK phosphorylation began as early as 10 min, increased between 20 and 60 min and slowly attenuated thereafter. IKK activation profiles were similar in cells exposed to TWS119. Furthermore, TWS119-pretreated BV-2 cells showed no differences from vehicle-treated control cells in LPS-induced IκB-α phosphorylation, degradation, and resynthesis. The resynthesized IκB-α was seen to be phosphorylated again by sustained IKK activation. This phenomenon has also been reported for LPS-challenged murine embryonic fibroblasts in which the sustained IKK activation is mediated by a TNF-α positive feedback mechanism following TLR4 signaling [32,33].

### Inhibition of GSK-3β reduces LPS-induced transcriptional activity of NF-κB

We next sought to determine the effects of inhibition of GSK-3β by TWS119 on downstream signaling of IKK activation and IκB-α degradation. Coincident with the LPS-elevated IKK activity and subsequent degradation of IκB-α in cells is the release of NF-κB for accumulation in the nucleus. Therefore, we addressed nuclear accumulation of p65 in BV-2 cells. Stimulation with LPS resulted in elevated levels of p65 in the nuclei of both control and TWS119-treated cells (Figure 3B). The nuclear levels of p65 in TWS119-pretreated cells were similar to controls. Additionally, levels of p65 decreased at a similar rate in the nuclei of both control and TWS119-pretreated BV-2 cells, suggesting no defects in either the rate of p65 nuclear entry or the rate of p65 export following GSK-3β inhibition by TWS119.

We further investigated whether TWS119 affects NF-κB p65 DNA binding activity by using an ELISA-based assay to measure LPS-induced DNA binding. The results show a marked increase in amount of p65 bound to consensus site oligonucleotides fixed to the ELISA plate following LPS stimulation (Figure 3C). Pretreatment with TWS119 had no effect on LPS-induced p65 DNA binding.

To assess whether the suppressive effect of TWS119 on TNF-α gene transcription is mediated by downregulating NF-κB transactivation, we transduced BV-2 cells with a reporter gene in which luciferase transcription is driven by three NF-κB consensus sites. LPS treatment alone prominently elicited transcriptional activity of the NF-κB consensus promoter in the transfected luciferase reporter gene. This effect was significantly attenuated by concomitant TWS119 treatment (Figure 3D). These findings confirm that the inhibitory effect of inactivated GSK-β on TNF-α gene expression is, at least partially, mediated by inhibition of NF-κB transactivation activity.

### GSK-3β inactivation inhibits NF-κB acetylation at Lys310 but not phosphorylation

**Figure 4:**
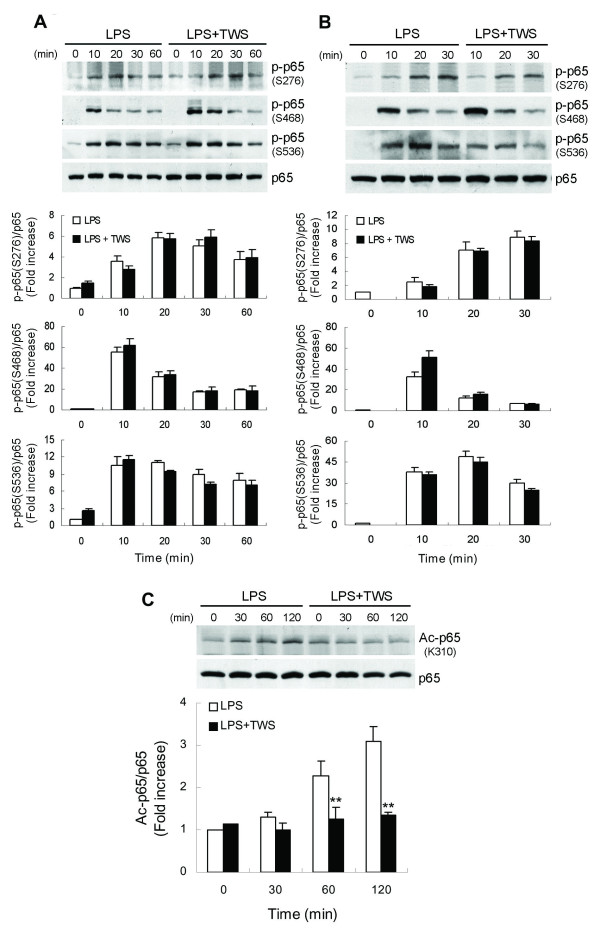
**GSK-3β inactivation inhibits NF-κB acetylation at Lys310 but not phosphorylation**. BV-2 cells (A) or primary microglia (B and C) were pretreated with vehicle or TWS119 (2.5 μM) for 30 min and then stimulated with LPS (100 ng/ml) for the indicated times. Whole cell lysates were prepared and subjected to western blotting using antibodies specific for phosphorylated (Ser276, 468 and 536), acetylated (Lys310) or total forms of p65. The immunoblots are representative of three independent experiments. Data are presented as mean ± SEM for three independent experiments. **p < 0.01 compared with respective cultures treated with LPS alone.

NF-κB activation, characterized by phosphorylation of specific amino acid residues in the p65 subunit, is one important prerequisite for transactivation of the target genes [7,34,35]. We included in our analysis the assessment of phosphorylation of p65. In control BV-2 cells or primary microglia, LPS stimulation resulted in enhanced phosphorylation of p65 at Ser276, 468 and 536 (Figure 4A, B). Cells pretreated with GSK-3β inhibitor TWS119 did not show compromised induction of phosphorylation at any of the three sites upon treatment with LPS.

In addition, NF-κB signaling is also modulated by post-translational modifications, including reversible acetylation of the p65 subunit [36]. Full transcriptional activity of p65 requires acetylation of Lys310 [37]. Using an antibody specific for acetylated Lys310, we found that LPS induced increased levels of acetylated p65 (Figure 4C). Treatment with TWS119 diminished levels of p65 with acetylated Lys310. These results suggest that inactivation of GSK-3β downregulates NF-κB activation, possibly by inhibiting acetylation of p65 on Lys310.

### GSK-3β inhibition blocks LPS-induced TNF-α production by inhibiting JNK signaling

**Figure 5:**
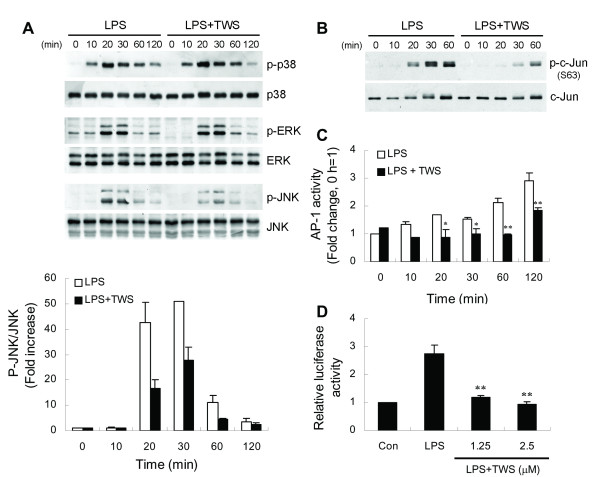
**Inhibition of GSK-3β activity blocks LPS-induced JNK signaling**. BV-2 cells were pretreated with vehicle or TWS119 (2.5 μM) for 30 min and stimulated with LPS (100 ng/ml) for the indicated times. Whole cell extracts or nuclear proteins were isolated. (A) Western analysis was used to determine LPS-induced p38, ERK and JNK activation in the absence or presence of TWS119. (B) Phosphorylated and total c-Jun was detected by western blotting in whole cell extracts. (C) ELISA-based measurement of c-Jun DNA binding was analyzed as described in *Methods*. Data are presented as mean ± SEM for three independent experiments. *p < 0.05; **p < 0.01 compared with respective LPS-only-treated cells. (D) Cells were transfected with a 3XAP-1-luciferase reporter construct. 24 h post-transfection, cells were preincubated with vehicle or various concentrations of TWS119 for 30 min before stimulation with 100 ng/ml LPS for 6 h. Luciferase activity is presented as a fold of control. Data are presented as mean ± SEM for three independent experiments. **p < 0.01 compared with LPS alone.

LPS is known to stimulate TNF-α production in microglia by activating MAP kinase signaling [38]. To investigate whether these kinases are modulated by GSK-3β, BV-2 cells were pretreated with TWS119 for 30 min followed by stimulation with LPS. Activation of three MAPKs, including p38, ERK and JNK, was analyzed by western blotting. As shown in Figure 5A, TWS119 significantly reduced the amount of activated JNK but not p38 MAPK and ERK.

As mentioned above, inhibition of GSK-3β by TWS119 inhibited LPS-induced phosphorylation of JNK. c-Jun is a component of the transcription factor AP-1 that binds and activates transcription at TRE/AP-1 elements. The transcriptional activity of c-Jun is regulated by phosphorylation at Ser63 and Ser73 [39]. The MAP kinase JNK binds to the amino-terminal region of c-Jun and phosphorylates c-Jun at Ser63/73 [40]. Therefore, we monitored phosphorylation of c-Jun in the same extracts used to study activation of the MAPKs. Figure 5B shows LPS-induced phosphorylation of c-Jun on Ser63 which correlates with the kinetics of JNK activation by LPS. Pretreatment of BV-2 cells with TWS119 abrogated LPS-evoked phosphorylation of c-Jun. Furthermore, it is well documented that NF-κB and AP-1 transcription factors play a major role in LPS-induced TNF-α production. First, we examined the effect of TWS119 on LPS-induced AP-1 DNA binding activity. Preincubation of cells with TWS119 decreased LPS-stimulated AP-1 binding (Figure 5C). Next, the effect of inhibition of GSK-3β on gene expression mediated by AP-1 was determined in BV-2 cells transfected with an AP-1 binding sites-containing reporter plasmid. As illustrated in Figure 5D, TWS119 abolished LPS-induced AP-1-dependent gene expression.

**Figure 6:**
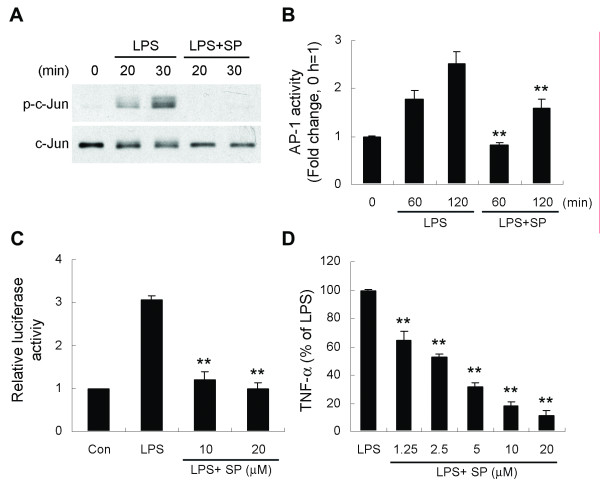
**SP600125, a JNK inhibitor, mimics GSK-3β inhibitor action in LPS-treated BV-2 cells**. Cells were pretreated with vehicle or SP600125 (SP, 20 μM) for 30 min followed by treatment with LPS (100 ng/ml) for the indicated times. Whole cell extracts or nuclear proteins were isolated. Western blotting (A) and ELISA-based measurement of c-Jun DNA binding (B) were analyzed as described in Fig. 5. (C) AP-1 luciferase reporter assays were performed by LPS stimulation in the absence or presence of SP600125 (6 h) of BV-2 cells. (D) Cells were pretreated with various concentrations of SP600125 for 30 min followed by exposure to 100 ng/ml LPS for 6 h. Released TNF-α was measured by ELISA. Data are presented as mean ± SEM for three independent experiments. **p < 0.01 compared with respective LPS-only-treated cells in (B) or compared with LPS alone in (C) and (D). The TNF-α content in untreated cultures was not detectable. The level of TNF-α in cells treated with LPS alone was 4.22 ± 0.25 ng/ml.

To further confirm whether JNK is a critical downstream signaling molecule in GSK-3β mediation of LPS-induced TNF-α production, BV-2 cells were pretreated with the JNK inhibitor SP600125 and then stimulated with LPS. We found that SP600125 treatment attenuated LPS-induced c-Jun phosphorylation, AP-1 DNA binding activity, and AP-1-dependent reporter gene expression (Figure 6A-C). Furthermore, LPS-induced TNF-α production was inhibited by SP600125 in a dose-dependent manner (Figure 6D).

### GSK-3β inactivation inhibits MLK3 signaling

**Figure 7:**
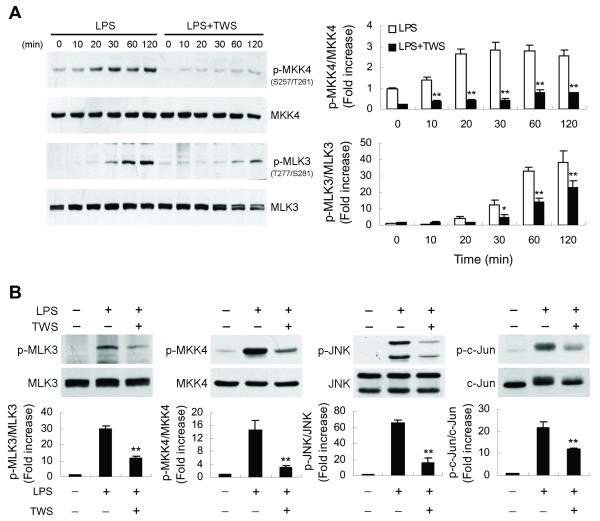
**Decreased GSK-3β activity blocks MLK3 signaling**. BV-2 cells (A) or primary microglia (B) were pretreated with vehicle or TWS119 (2.5 μM) for 30 min and then stimulated with LPS (100 ng/ml) for the indicated times (A) or 30 min (B). Western analysis was used to determine total and phosphorylated c-Jun, JNK, MKK4 and MLK3 proteins in whole cell extracts. Data are presented as mean ± SEM for three independent experiments. **p < 0.01 compared with respective cultures treated with LPS alone.

**Figure 8:**
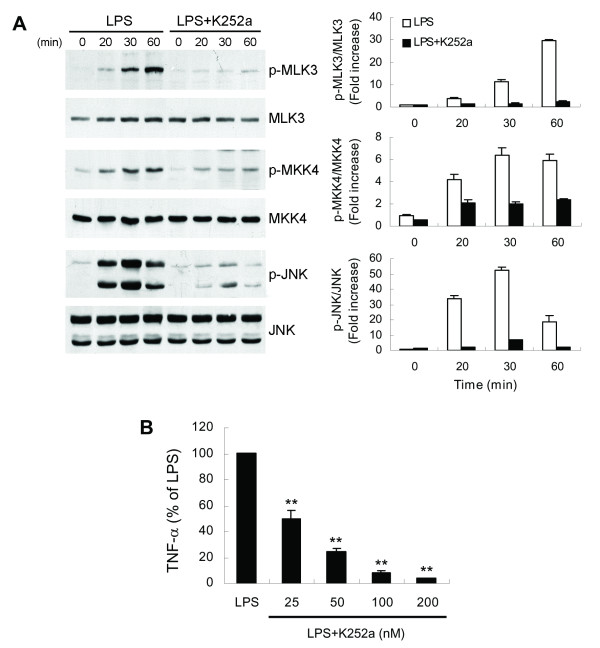
**The MLK3 inhibitor K252a prevents LPS-induced MLK3/JNK signaling cascades and TNF-α production**. BV-2 cells were pretreated with vehicle or 100 nM (A) or various concentrations (B) of K252a for 30 min followed by treatment with 100 ng/ml LPS for the indicated times (western blotting) or 6 h (TNF-α). Western analysis was performed as described in Fig. 7. Released TNF-α (B) was measured by ELISA. Data are presented as mean ± SEM of three independent experiments. **p < 0.01 compared with LPS-stimulated alone cultures. The TNF-α content in untreated cultures was not detectable. The level of TNF-α in cells treated with LPS alone was 4.26 ± 0.14 ng/ml.

To provide further insight into the regulatory role of GSK-3β in JNK signaling cascades, we investigated the effect of this enzyme on upstream kinases of the JNK pathway. JNK activation is regulated by two upstream mitogen-activated protein kinase (MAPK) kinases, MKK4 and MKK7 [41,42]. The results show that LPS treatment failed to cause MKK7 phosphorylation (data not shown), whereas a lasting activation of the constitutively present MKK4 was induced (Figure 7A). Pretreatment of cells with TWS119 led to suppression of LPS-induced MKK4 phosphorylation. Mixed lineage kinase 3 (MLK3) is characterized as a MAPK kinase kinase that activates the JNK pathway through dual phosphorylation of MKK4/7 [43,44]. To further determine whether MLK3 is inhibited by GSK-3β inactivation, the same samples were then examined for MLK3 phosphorylation using a phospho-specific antibody that detects the autophosphorylation status of MLK3 at Thr277 and Ser281, residues necessary for MLK3 kinase activity that are located within the kinase domain [45]. Figure 7A shows that LPS induced a time-dependent increase in MLK3 autophosphorylation and that TWS119 prevented this phosphorylation. Similar findings were observed with LPS-stimulated primary microglia, where decreasing GSK-3β activity inhibited MLK3/JNK signaling (Figure 7B). We next used the MLK3 inhibitor k252a, which inhibits MLK3 by competing with ATP [46,47], to investigate the role of MLK3 in the GSK-3β inactivation-mediated decrease in TNF-α production. LPS-induced MLK3 autophosphorylation in BV-2 cells was markedly abolished by k252a (Figure 8A). In addition, k252a also blocked the LPS-induced downstream phosphorylation of MKK4 and JNK, leading to suppression of TNF-α release (Figure 8A, B).

**Figure 9:**
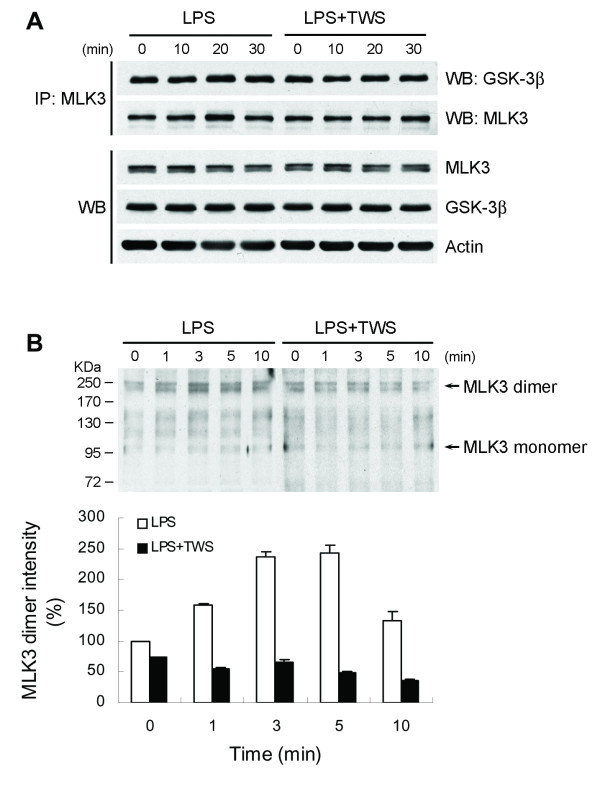
**Association of MLK3 and GSK-3β**. BV-2 cells were pretreated with vehicle or TWS119 (2.5 μM) for 30 min prior to LPS treatment for the indicated times. Cell lysates were immunoprecipitated (IP) with goat anti-MLK3 antibody, and the resulting precipitates were separated by SDS-PAGE in the presence (A) or absence (B) of the reducing agent DTT. Immunoblotting was performed using rabbit anti-GSK-3β antibody or rabbit anti-MLK3 antibody. The cell extracts were also blotted for MLK3, GSK-3β and β-actin. The band intensity of MLK3 dimers was quantified with a densitometric analysis, and calculated as the optical density × area of band.

As mentioned above, MLK3 activity was blocked by a GSK-3β-selective inhibitor, as indicated by reduced phosphorylation of MKK4, suggesting that GSK-3β lies upstream of MLK3. GSK-3β appears to inhibit the autophosphorylation activity of the MLK3 kinase domain even though the MLK3 kinase domain is not phosphorylated by GSK-3β [48]. For this reason, the interaction of endogenous MLK3 and GSK-3β was examined by coimmunoprecipitation. Immunoprecipitation of cell lysates with anti-MLK3 antibody resulted in specific coimmunoprecipitation of GSK-3β with MLK3 (Figure 9A). This interaction was not markedly affected by exposure of the cells to LPS. Furthermore, neither the amount nor the duration of GSK-3β association was affected after stimulation with TWS119. Dimerization of MLK3 has been shown to be a prerequisite for its autophosphorylation and, thereby, activation [49]. To determine whether MLK3 dimerization is disrupted by inhibiting GSK-3β activity, we used coimmunoprecipitation and nonreducing SDS-PAGE to determine the disulfide-linked MLK3 dimer [49]. When separated by SDS-PAGE under nonreducing conditions, the disulfide bonds of these protein dimers are preserved and can be detected as protein complexes migrating at approximately twice the size of the corresponding monomeric form. As shown in Figure 9B, in the absence of the reducing agent DTT, both monomeric (of 105 KDa) and dimeric (of approximately 220 kDa) forms of MLK3 was observed. Exposure of cells to LPS resulted in an increase in MLK3 dimers, whereas inactivation of GSK-3β by TWS119 blocked MLK3 dimerization.

### The interactions of GSK-3β-mediated NF-κB and MLK3/JNK pathways

**Figure 10:**
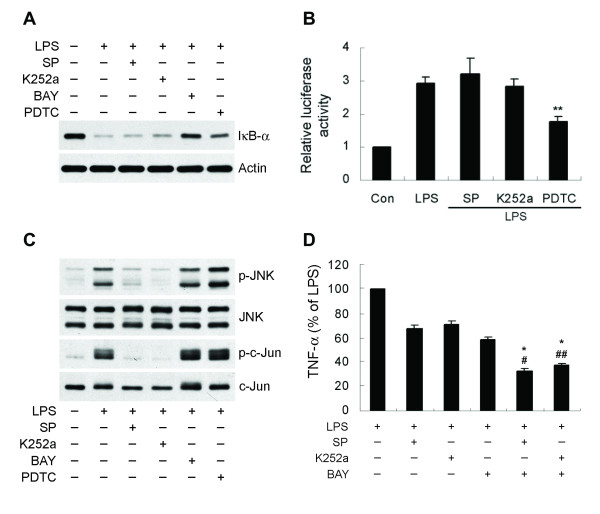
**GSK-3β-mediated NF-κB and MLK3/JNK are two independent signaling pathways in the induction of TNF-α**. (A) BV-2 cells were pretreated with SP600125 (SP, 10 μM), K252a (50 nM), BAY 11-7082 (BAY, 5 μM) or PDTC (20 μM) for 30 min following exposure to 100 ng/ml LPS for another 30 min. IκB-α degradation was determined by western blotting. (B) NF-κB luciferase reporter assays were performed by LPS stimulation in the absence or presence of 10 μM SP600125, 50 nM K252a or 20 μM PDTC (6 h) of BV-2 cells as described in Fig. 3. Data are presented as mean + SEM for three independent experiments. **p < 0.01 compared with LPS alone. (C) Cells were treated as described in (A). Phosphorylated and total JNK and c-Jun were detected by western blotting in whole cell extracts. (D) Cells were preincubated with SP600125 (2.5 μM), K252a (12.5 nM), BAY 11-7082 (0.625 μM), SP600125 plus BAY 11-7082 or K252a plus BAY 11-7082 for 30 min, and then treated with 100 ng/ml LPS for 6 h. Released TNF-α was measured by ELISA. Data are presented as mean ± SEM for three independent experiments. #*p*, ##*p *or *p < 0.01 compared with SP600125, K252a or BAY 11-7082 treated cells, respectively. The TNF-α content in untreated cultures was not detectable. The level of TNF-α in cells treated with LPS alone was 5.74 ± 0.41 ng/ml.

As mentioned above, both the LPS-activated NF-κB and the MLK3/JNK signaling cascades are mediated by GSK-3β. However, in activated microglia the interactions of these two pathways are not well understood. We therefore examined the relationship between NF-κB and MLK3/JNK in the signaling of GSK-3β following treatment of microglia with LPS. As shown in Figure 10, neither a MLK3 inhibitor, K252a nor a JNK inhibitor, SP600125 had any effect on LPS-induced IκB-α degradation (Figure 10A) or NF-κB transcriptional activity (Figure 10B). In addition, neither BAY 11-7082 and PDTC, two NF-κB inhibitors, significantly altered levels of JNK or c-Jun phosphorylation (Figure 10C). Treatment with a combination of an MLK3/JNK inhibitor and an NF-κB inhibitor showed an additive inhibitory effect on TNF-α induction compared with each treatment alone (Figure 10D). These data indicate that GSK-3β-mediated the NF-κB and MLK3/JNK signaling pathways independently lead to induction of TNF-α in LPS-stimulated microglia.

## Discussion

In the present study, we have demonstrated that treatment of microglia with either selective GSK-3β inhibitors or small interfering RNA targeting GSK-3β inhibits TNF-α secretion induced by LPS stimulation. This investigation of the central mechanism by which GSK-3β positively regulates the inflammatory response showed that GSK-3β inactivation suppresses TNF-α production by inhibiting NF-κB p65 transactivation activity through deacetylation of p65 at Lys310. In addition, we also found that prevention of MLK3/JNK signaling cascades is another essential mechanism responsible for GSK-3β inhibition-mediated anti-inflammatory actions.

GSK-3β has been shown to play a critical role in inflammatory processes [11,50]. We herein examined the role of GSK-3β in modulating microglial inflammatory responses. Using pharmacological approaches, we found that inhibition of GSK-3β can significantly inhibit LPS-induced TNF-α production in microglia. Furthermore, treatment of BV-2 microglia with siRNA targeting GSK-3β can block TNF-α release. In the present study, BV-2 microglial cells appear to be more responsive to GSK-3β inhibitor treatment than are primary microglial, based on reduction in TNF-α levels. Whether this difference in sensitivity is due to differential intracellular activity of inhibitor or divergent response to GSK-3β inhibition in these two cell types needs further investigation. Our data are consistent with previous reports showing that GSK-3β positively regulates expression of pro-inflammatory genes in LPS-stimulated human monocytic cells and mouse hippocampal slice cultures [11,13,51]. However, Vines et al. [10] have shown that overexpression of GSK-3β in endothelial cells significantly inhibits TNF-α- and IL-1β-induced expression of IL-6, monocyte chemoattractant protein 1, and vascular cell adhesion molecule 1. A previous report demonstrated that inhibition of GSK-3β enhances LPS induction of TNF-α expression in cardiomyocytes [9]. These findings indicate that the role of GSK-3β in inflammatory responses may depend on cell type.

Huang et al. [12] have shown that inhibition of GSK-3 reduces LPS-induced NO and RANTES production by triggering anti-inflammatory IL-10 upregulation in microglia. However, our study demonstrates that blocking endogenous IL-10 effects by using an anti-IL-10 antibody has no effect on the effects of GSK-3β inhibitors in reducing TNF-α release (data not shown), suggesting that GSK-3β inactivation-mediated decrease of TNF-α occurs through a distinct mechanism. NF-κB is a pluripotent nuclear transcription factor implicated in the regulation of multiple cellular processes, including the inflammatory response. A growing body of evidence suggests that GSK-3β is critically involved in NF-κB signaling transduction and is essential for NF-κB activation [52-54]. Recent evidence suggests that inactivation of GSK-3β only affects downstream events of NF-κB activation, since upstream events like IκB-α phosphorylation and degradation and nuclear accumulation of NF-κB are barely altered by GSK-3β inhibition [11,55]. This is again confirmed in our present study in microglia. However, our data regarding the role of GSK-3β in LPS-induced cytoplasmic signal transduction pathways differ significantly from those of some reports and suggest cell type-specific functions and stimulus for GSK-3β. Using TNF-α-treated primary astrocytes, Sanchez et al. [6] have shown that GSK-3β downregulates IKK activity, stabilizes IκB-α, and prevents p65 accumulation in nuclei. Another study showed that genetic deletion of GSK-3β abrogates activation of a number of cytoplasmic signaling intermediates and nuclear translocation of p65 [8]. Although the early steps leading to NF-κB activation were unaffected by inactivation of GSK-3β, our results show that GSK-3β inhibition attenuates p65-dependent transcription, suggesting that GSK-3β positively regulates NF-κB in LPS-stimulated microglia through reduction of transactivation activity of p65.

Once activated, NF-κB transcriptional activity is further regulated by inducible post-translational modifications, including phosphorylation and acetylation [37,56-58]. A number of different phosphorylation sites have been identified on the p65 subunit. This phosphorylation is essential for NF-κB nuclear transportation, subunit dimerization, DNA binding, and finer regulation of NF-κB transcriptional activity [7,34,35,57]. Therefore, one possible mechanism by which GSK-3β may control LPS-induced NF-κB activity may be through direct phosphorylation of NF-κB. Exposure of microglia to LPS resulted in serine phosphorylation at 276, 468, and 536 sites in p65. However, inhibition of GSK-3β had no suppressive effect on phosphorylation of all three sites. A previous report implicated GSK-3β in phosphorylation of p65 at Ser468 and demonstrated that this regulates basal levels of p65 transactivation in HeLa cells [7]. Gong et al. [59] reported that GSK-3β inactivation downregulates NF-κB activity via inhibition of p65 phosphorylation at Ser468 in TNF-α-treated renal tubular epithelial cells. Our study found no reduction in Ser468 phosphorylation in microglia pretreated with GSK-3β inhibitor. Possible interpretations of our findings are that GSK-3β phosphorylates p65 at Ser468 in a cell type-specific manner or that Ser468 phosphorylation under some conditions is mediated by a multikinase complex.

The nuclear function of the heterodimeric NF-κB transcription factor is regulated in part through reversible acetylation of its p65 subunit. Site-specific acetylation of p65 regulates discrete biological actions of the NF-κB complex [37,60]. Acetylation of lysine 310 has been shown to be required for full transcriptional activity of p65. In the present study, stimulation of microglia with LPS increased acetylation of p65 at lysine 310, and the addition of a GSK-3β inhibitor decreased levels of acetylated p65, suggesting that GSK-3β inhibition-mediated downregulation of NF-κB transcriptional activity might be, at least partially, attributable to decreased p65 acetylation at lysine 310. In the nucleus, p65 associates with p300/CBP transcriptional co-activators. The acetyltransferases p300 and CBP appear to play a major role in the *in vivo *acetylation of p65 [37,61]. A prior study demonstrated that GSK-3β inhibition suppresses the binding of NF-κB p65 to the nuclear co-activator CBP [11]. This is in agreement with our work showing that acetylation of p65 at lysine 310 is suppressed by inactive GSK-3β. The phenomenon that inhibition of GSK-3β impairs acetylation of proteins also occurs for the tumor suppressor protein p53 [62]. Our data provide a molecular understanding of how GSK-3β inhibition suppresses NF-κB-mediated production of TNF-α in LPS-stimulated microglia.

LPS stimulation of microglia activates all three MAPK pathways: p38, ERK1/2 and JNK [38,63]. Investigation of the mechanism by which GSK-3β positively regulates LPS-induced TNF-α demonstrated that loss of GSK-3β activity abrogated LPS-induced activation of JNK, leading to decreased c-Jun phosphorylation and AP-1 activation. Pharmacologically, we further demonstrated that SP600125, a JNK inhibitor, exhibited a similar ability to block AP-1 activation and TNF-α production. Studies on the role of GSK-3β in JNK activation are controversial. One previous study showed that homozygous disruption of GSK-3β dramatically sensitizes mouse embryonic fibroblasts to JNK activation induced by lysophosphatidic acid and sphingosine-1-phosphate [64]. On the contrary, GSK-3β has also been shown to be a positive regulator of TNF-induced activation of JNK [8]. Furthermore, inhibition of GSK-3β by lithium has no effect on LPS-induced JNK activation in human monocytic cells [51]. These differences may reflect the use of different cell types. The present data is the first to show a positive regulatory role for GSK-3β in LPS-induced JNK activation in microglia, suggesting that the reduction of LPS-induced TNF-α by GSK-3β inactivation is due, at least in part, to inhibition of the downstream signaling molecule JNK.

In the JNK pathway, activating stimuli activate MAP3K members, such as MLK3 or MEKK1, which in turn phosphorylate MAP2K members such as MKK4 and MKK7. The activated MAP2Ks then phosphorylate JNK. It has been reported that GSK-3β physically associates with and activates MEKK1 under either basal or UV- or TNF-α-stimulated conditions, thereby stimulating the JNK pathway [65]. The present data demonstrates that MLK3 inhibitor markedly inhibites JNK activation, indicating that, in microglia, the activation of JNK in response to LPS is mediated via MLK3. MLK3 autophosphorylation (Thr277 and Ser281) has been shown to correlate tightly with activity [45]. Our results reveal that attenuation of LPS-induced MLK3 autophosphorylation by decreasing GSK-3β activity prevents a stress kinase cascade that leads to inactivation of JNK. To our knowledge, there is no prior report about the effect of GSK-3β on autophosphorylation of MLK3. This suggests that GSK-3β inhibition abrogates LPS-induced activation of JNK due, at least partially, to a lack of activation of MLK3. Dimerization-induced autophosphorylation within the kinase domain has been shown to be essential for activation of MLK3 [49]. Our findings show that GSK-3β physically interacts with MLK3 and inactivation of GSK-3β results in decreased MLK3 dimerization, indicating that this association induces activation of MLK3 also through a mechanism independent of direct protein phosphorylation by GSK-3β [48]. Taken together, our results provide the novel information that GSK-3β is a potent upstream activator of MLK3 in the LPS-induced TNF-α production pathway.

## Conclusion

Decreasing GSK-3β activity downregulates the transactivation efficiency of NF-κB by inhibiting p65 acetylation, and blocks the MKK4/JNK pathway by disrupting MLK3 dimerization-induced autophosphorylation, ultimately leading to attenuation of TNF-α production in LPS-stimulated microglia. Because of the critical roles of NF-κB and JNK/AP-1 in neuroinflammation induced by a variety of stimuli, and because GSK-3β inhibition enables simultaneous regulation of multiple transcription factors involved in inflammatory signaling [66], one could postulate that GSK-3β might provide a potential target for anti-inflammatory intervention. Downregulation of microglia-mediated inflammation by impairing GSK-3β to prevent neuronal degeneration requires further in vivo investigation.

## Competing interests

The authors declare that they have no competing interests.

## Authors' contributions

MJW, HYH, WFC and HFC carried out the experimental work. JSK provide useful advice and reviewed the manuscript. MJW contributed to the design, analysis of the data, and manuscript preparation. All of the authors have read and approved the final version of the manuscript.
